# General Commentary: From hospital exemption to evidence: a statistical framework for leveraging real-world data

**DOI:** 10.3389/fimmu.2026.1868957

**Published:** 2026-07-31

**Authors:** Gloria Brigiari, Maurizio Muraca, Dario Gregori

**Affiliations:** 1 Unit of Biostatistics, Epidemiology and Public Health, Department of Cardiac, Thoracic, Vascular Sciences and Public Health, University of Padova, Padova, Italy; 2 BIOSTAT-X Biostatistics & AI for Biomedical Discovery, Institute of Pediatric Research (IRP) “Città della Speranza”, Padova, Italy; 3 PhD Program in Translational Specialistic Medicine “G.B. Morgagni”, Curriculum “Biostatistics and Clinical Epidemiology”, University of Padova, Padova, Italy; 4 Institute of Pediatric Research “Città della Speranza”, Padua, Italy

**Keywords:** ATMP, Bayesian borrowing, hospital exemption, pediatric rare disease, RWE

## Introduction

We read with great interest the report by Ussowicz et al. (1), which describes the use of donor-derived virus-specific T lymphocytes (VSTs) administered as hospital-exemption advanced therapy medicinal products (ATMP-HE) to six pediatric transplant recipients with refractory viral infections. Their work provides valuable early real-world evidence on VST feasibility and antiviral activity in a vulnerable population, while also documenting the limitations of outcome data generated outside a formal trial framework. In our view, these findings invite constructive discussion beyond VST immunotherapy and speak directly to the urgent need for a statistical and methodological infrastructure capable of transforming HE-generated patient data into rigorous evidence for pediatric ATMP development.

## Small cohorts and structured evidence borrowing

The small-cohort problem is precisely where modern statistical methods can help. Ussowicz et al. treated six patients across two Polish centers, with virological response evaluable in only five. The cohort was heterogeneous in underlying disease, transplant type, viral target, and timing of VST infusion, limitations that the authors appropriately acknowledge. Yet this scenario—small, heterogeneous, single-arm, observational—is not an anomaly: it is the defining feature of HE-based ATMP use in pediatric rare diseases. Ambrosone and Cometa recently documented that across a full decade of Italian HE experience, 329 ATMP-HE treatments were authorized for conditions such as neuroblastoma, B-ALL, and ADA-SCID, but follow-up data were received for only 119 patients (37%) (2). Neller et al. reported 15 years of compassionate-access VST use under the Australian TGA Special Access Scheme, including 78 treated patients from 115 requests across hospitals in Australia and New Zealand, targeting EBV, CMV, BKV/JCV and AdV; however, the analysis remained retrospective and largely descriptive (3). Individually, each cohort is too small and too heterogeneous for classical frequentist inference. Collectively, however, these experiences represent a substantial and growing body of evidence that remains methodologically untapped.

Bayesian statistical frameworks offer a principled solution. Meta-Analytic-Predictive (MAP) priors, commensurate priors, and related borrowing approaches can incorporate external data while down-weighting sources that conflict with the current study population (4, 5). Rare-disease trial literature similarly emphasizes the need to leverage auxiliary data and to evaluate trial designs through simulation (6). For HE-generated ATMP data, borrowing should therefore be conservative and pre-specified: protocols should define eligible external sources, endpoint definitions, commensurability criteria, prior weights or effective-sample-size caps, and sensitivity analyses before HE evidence is used to inform a trial.

## Data quality, harmonization, and interoperability

The value of any borrowing framework depends entirely on data quality and interoperability. Ussowicz et al. note that immunological monitoring was not standardized and that antigen-specific T-cell enumeration was not available in their HE experience (1). This is not a criticism of the authors; it reflects the clinical urgency of HE use. However, it also exposes the fundamental bottleneck. HE protocols should prospectively define a minimum dataset, including baseline disease and transplant context, viral target and viral load, VST source, dose, timing and HLA match, concomitant antivirals and immunosuppression, virological and clinical endpoints, immunological monitoring, adverse-event grading using CTCAE for general events, ASTCT criteria for CRS/ICANS where relevant, standard GVHD grading, and harmonized follow-up intervals. Alignment with interoperable standards such as CDISC and OMOP CDM, and with FAIR principles, would support cross-center pooling through rare-disease registries and European Reference Network infrastructure (7–9).

## Formalizing the hospital-exemption-to-trial bridge

The ALLOVISTA framework itself illustrates the HE-to-trial bridge that the field needs to formalize. Ussowicz et al. describe six patients treated under HE within the broader organizational framework of a registered Phase I trial. This dual-track model—HE for patients who cannot wait, formal trial for systematic dose escalation—is pragmatically sound but statistically ad hoc. A pre-specified Bayesian borrowing protocol would formalize the relationship: once the posterior distribution from accumulated HE evidence crosses an informativeness threshold, the adaptive trial can incorporate HE-derived priors, substantially reducing the sample size needed for dose-finding or preliminary efficacy assessment while maintaining rigorous error control (5, 6). The trajectory of ARI-0001 in Spain illustrates how academic ATMP development can move from clinical testing to national hospital-exemption authorization, providing a bridge toward broader clinical access (10).

## Discussion

This discussion belongs within the substantial body of European regulatory guidance already addressing evidence generation in small and paediatric populations. The EMA Guideline on Clinical Trials in Small Populations recognises the constraints of limited sample sizes and endorses methodological responses, including Bayesian approaches and the use of external and historical data (11). Paediatric development is further supported by a formalised extrapolation framework, articulated in the ICH E11A guideline, providing a principled basis for borrowing evidence across populations when biological and clinical assumptions are justified (12). Sustained regulatory effort is also directed at qualifying real-world data for regulatory purposes, with the statistical challenges themselves recognised in this evolving guidance (13). These frameworks are not merely aspirational: conditional marketing authorisation and authorisation under exceptional circumstances already accommodate decision-making under residual uncertainty where comprehensive data cannot reasonably be generated, and are expected to persist under the reformed legislation (14, 15). The authorisation of onasemnogene abeparvovec (Zolgensma) for spinal muscular atrophy type 1 is illustrative: evidence was generated from a single-arm study in which patients were enrolled directly into a treatment arm, with survival and motor outcomes assessed against natural-history cohorts (16). This precedent underscores that the infrastructure we advocate for HE-generated data builds upon, rather than departs from, an established tradition of accommodating small-population evidence.

Looking ahead, the proposed EU pharmaceutical legislation reform provides for strengthened HE data collection and EMA-level transparency (17), and the EPTRI/EAHP/EUEYE/SIOPE joint position paper advocates formalizing HE cases as supporting safety information for clinical trial entry (18). Ussowicz et al.’s experience in Poland, alongside Italy’s decade of HE data and Spain’s academic CAR-T ARI-0001 programme authorized under hospital exemption, demonstrates both the clinical value and the evidential fragility of HE-generated outcomes. What remains missing is the statistical bridge: a validated, regulatory-acceptable methodology for incorporating HE real-world evidence into the formal evidentiary architecture of pediatric ATMP development. Building this bridge—through harmonized minimum datasets, Bayesian borrowing frameworks, and cross-border registry infrastructure—is the essential next step to ensure that every child treated under hospital exemption contributes not only to their own care but to the evidence base that will benefit future patients.
